# Risk factors of sleep paralysis in a population of Polish students

**DOI:** 10.1186/s12888-022-04003-0

**Published:** 2022-06-07

**Authors:** Paulina Wróbel-Knybel, Michał Flis, Joanna Rog, Baland Jalal, Hanna Karakuła-Juchnowicz

**Affiliations:** 1 I Department of Psychiatry, Psychotherapy and Early Intervention, Medical University of Lublin, Głuska 1 Street, 20-439 Lublin, Poland; 2 Department of Psychology, Harvard University, 33 Kirkland St, Cambridge, MA 02138 USA; 3 Department of Psychiatry, University of Cambridge, Downing Street, Cambridge, CB2 3EB UK

**Keywords:** Sleep paralysis, Anxiety, Parasomnia, Sleep disorder, PTSD, Health status, Sport, Mental disorder, Sleep, Worry

## Abstract

**Background:**

Sleep paralysis (SP) is a transitional dissociative state associated with the REM sleep phase that affects approximately 28.3% of the student population during their lifetime. The reasons for the high prevalence of SP in the student population are not entirely clear. Research indicates possible influencing factors such as the intensification of anxiety symptoms, a tendency to worry, the presence of PTSD symptoms, and behavioral factors such as the consumption of psychoactive substances (caffeine, alcohol, nicotine), sleep deprivations and poor sleep hygiene. The study aimed to assess the prevalence of SP and determine the risk factors for the occurrence of SP in the population of Polish students.

**Methods:**

The study used a battery online consisting of a set of questionnaires 1) a personal questionnaire, 2) the SP-EPQ, 3) the PCL −5, 4) the STAI-T, 5) the PSWQ. The questionnaire was sent via Facebook to 4500 randomly selected students from different universities in Poland. The questionnaire was completed by 2598 students. To unify the participant sample, people over 35 were excluded from the study (45 students). Ultimately, data from 2553 students were analyzed.

**Results:**

A total of 33.14% of individuals experienced at least one episode of SP in their lives. The highest odds ratio for SP was associated with: the presence of three or more health problems (OR: 2.3; *p* = 0.002), the presence of any mental disorder (OR: 1.77; *p* = 0.002), including mood disorders (OR: 2.07; *p* = 0.002), suffering from at least one somatic disease (OR: 1.34; *p* = 0.002), a high level of anxiety as a constant personality trait (OR: 1.20; *p* = 0.035) and smoking (OR: 1.48; *p* = 0.0002), alcohol consumption (OR: 1.52; p < 0.0001), physical activity (OR: 1.31; *p* = 0.001).

**Conclusions:**

The results of our research indicate that a large proportion of students experienced isolated sleep paralysis. Mental and somatic health problems and lifestyle factors were found to predispose individuals to this disorder. Due to the numerous risk factors for SP, it is necessary to conduct additional research to confirm the impact of these factors and to investigate the mechanisms of their influence on SP.

**Supplementary Information:**

The online version contains supplementary material available at 10.1186/s12888-022-04003-0.

## Background

Sleep paralysis (SP) is a transitional dissociative state that occurs upon falling asleep or going from sleep to wakefulness [1, 2]. It usually lasts from a few seconds to several minutes, during which the person experiencing SP remains aware “is awake”, while the activity of the motor muscles is inhibited, which causes body paralysis [3–5]. The intercostal and oculomotor muscles usually retain their functions [6]. This disorder often occurs while sleeping on the back [7]. It is commonly associated with hypnopompic or hypnogogic hallucinations, and a feeling of excessive anxiety and fear [8–10]. However, hallucinations are not a necessity for the diagnosis of SP. The main and essential criterion that must be met to diagnose an episode of sleep paralysis is the presence of muscle atonia while maintaining consciousness [11].

The term Isolated Sleep Paralysis (ISP) is used when the episode of SP is not caused by other sleep disturbances, substances use, and physical or mental disorders. Recurring SP episodes accompanied by clinically significant anxiety and stress are classified as Recurrent Isolated Sleep Paralysis (RISP) [11]. SP episodes with markedly increased anxiety are called Fearful Isolated Sleep Paralysis (FISP) [8].

The pathomechanism of this disorder is not fully understood, but it is known that it is associated with an abnormal overlapping of the REM sleep phase with the waking state [6]. The studies conducted so far on this phenomenon show that there may be many predictors and the very pathophysiology of its occurrence is multifactorial [7, 12, 13].

Approximately 7.6% of the general population of the world has experienced at least one episode of SP [14]. Gender cannot be considered a risk factor because its prevalence is similar among women and men [13, 15–20]. The studies also do not reveal any relationship between the occurrence of SP and age [13, 15, 16, 21, 22]. However, many studies show that this disorder is more common in college students. A systematic review by Sharpless and Barber (2011) analyzed 35 studies on SP and reported that 28.3% of students experienced at least one SP episode [14]. The reasons for the high prevalence of SP in the student population are not entirely clear. Research indicates possible influencing factors such as the intensification of anxiety symptoms, a tendency to worry, the presence of PTSD symptoms, and behavioral factors such as the consumption of psychoactive substances (caffeine, alcohol, nicotine), sleep deprivations, and poor sleep hygiene [19, 23–25].

It is a fact that SP affects those more often who suffer from mental disorders [7, 14, 18]. Research shows that people suffering from anxiety disorders such as Generalized Anxiety Disorder (GAD), where SP prevalence reaches 15.8%, Panic Disorder, where it ranges from 20.8 to 30.6%, and Social Phobia 22%, are particularly vulnerable [18]. Many studies reveal an association of SP with Post-Traumatic Stress Disorder (PTSD) [10, 26], and traumatic experiences [13, 27–29].

The relationship between SP and the use of psychoactive substances such as caffeine, alcohol, and nicotine is not clear and has not been fully investigated [30]. Despite the well-known effect of caffeine on sleep quality, the studies conducted so far have not confirmed its role as a predictor of SP [17]. However, some reports support the relationship between drinking alcohol, smoking, and the occurrence of SP [13, 17, 19, 24].

The influence of sleep hygiene variables has been raised in many studies on sleep paralysis. It is known that SP can be caused by abnormal sleep habits [19, 24, 31]. It may also coexist with other sleep disorders, such as narcolepsy [32], nightmares, obstructive sleep apnea syndrome, exploding head syndrome, and in people who suffer from sleep without regeneration [19, 21, 31, 33–35]. However, so far, no association has been found between SP and insomnia, sleepwalking, idiopathic hypersomnia, or movement disorders during sleep [17, 33].

Other factors that may influence the occurrence of SP also include a high body mass index (BMI) [8], the level of general health [21, 36, 37], arterial hypertension [36], and chronic pain [22].

The student lifestyle is burdened with particular disharmony which is usually associated with adaptation to adult life in a new specific environment. Students must learn independent functioning whilst dealing with requirements related to a rapid acquisition of knowledge which leads to stress and an eventual irregular sleep cycle [38]. Additionally, improper eating habits, insufficient physical activity, family and relationship problems, financial woes, and difficulties in coping with the stress of meeting expectations are all factors to be taken into account when considering the general health of young scholars [39]. With this in mind, we expect that SP incidence rates in polish student populations would be associated with both the state of health and lifestyle factors.

This article is a continuation of our research on SP in the Polish population. So far, one study has been published involving a group of 439 students living in the Lubelskie Voivodeship [40]. The current study includes a population of 2553 students residing throughout Poland. The aim of the study was to: 1) assess the prevalence of SP; 2) identify potential factors which could be predictors of SP occurrence in a Polish student population.

## Methods

### Study participants and procedure

**Fig. 1:**
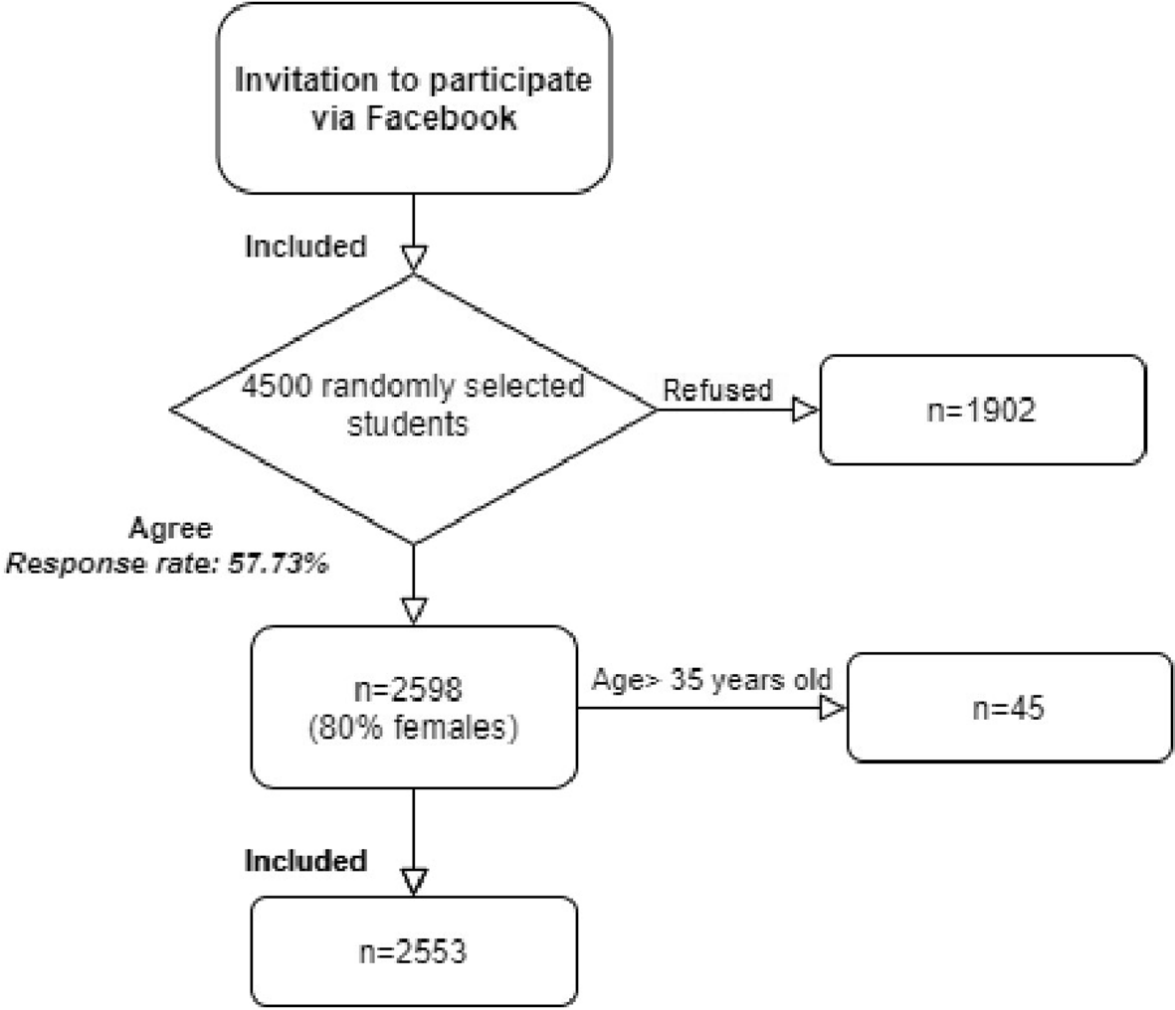
Study participants and procedure: a flowchart to procedure recruiting participants to study

This web-based cross-sectional survey was conducted from March 2018 to June 2018. The participants of the research were 4500 randomly selected students from various Polish universities (and different grades of study), who were sent a research survey via Facebook. The survey was completed by 2598 participants (response rate 57.73%): 2073 female students (80%) and 525 male students (20%) aged 18 to 50. To unify the participant sample, people over 35 were excluded from the study (45 students). The procedure of recruiting participants to study is shown in Fig. 1.

All the students participating in the research completed a battery of online questionnaires containing open-ended and closed-ended questions: 1) the proprietary personal questionnaire, 2) the Sleep Paralysis Experience and Phenomenology Questionnaire (SP-EPQ) [41], 3) the PTSD checklist (PCL) [42], 4) the State and Trait Anxiety Inventory (STAI-T) [43] and 5) the Penn State Worry Questionnaire (PSWQ) [44].

## Methods

### Personal questionnaire

In this study, we applied a modified version of our proprietary questionnaire, which we used in previous research [40, 45]. The questionnaire consists of the following parts:

I. personal data, i.e. gender, age, height, body mass, size of the city of residence, name of the University
II. lifestyle data, i.e. smoking (number of cigarettes smoked during the day and how long has the person been smoking in years), the average sleep duration during the day (number of hours), alcohol consumption (type of alcohol and frequency of alcohol drinking during the month), caffeine intake (number of cups of coffee during the day), physical activity (the number of hours of physical activity during the week).
III. health data, i.e. the presence of chronic diseases confirmed by a formal diagnosis (somatic and psychiatric) and medications taken.

### Sleep Paralysis Experience and Phenomenology Questionnaire (SP-EPQ)

We used the SP-EPQ questionnaire designed by Baland Jalal and Devon Hinton. It assesses the frequency, duration, and symptomatology of SP episodes (emotions, hallucinations, and psychosomatic symptoms), and other variables related to SP episodes (triggers, time of occurrence, sleeping position during SP episodes, and the level of knowledge about the phenomenon) [41]. Hitherto, this version of the questionnaire has been used in SP studies in Italy, Turkey, and Poland [40, 45, 46, 47,]. As part of the translation procedure into Polish conditions, the procedure of translation and retranslation by a certified translator from English into Polish was performed; followed by another translation by a professional translator, and then edited by a native speaker with medical education [40].

The SP-EPQ consists of 17 questions regarding SP episodes. The first question of the questionnaire is worded as follows: “Some people have experienced an incident where they couldn’t move their arms, legs, or speak while sleeping or waking even though they wanted to do so: Have you ever experienced this for yourself?” All of the twelve open-ended questions of the questionnaire are formulated in the same way, which allows for a verification of whether participants understand the questions as intended. The remaining five are closed-ended questions. Item 8 of the questionnaire comprises twelve closed questions regarding the presence of psychosomatic SP symptoms (somatic sensations) [40, 45–47]. The Cronbach’s alpha coefficient for this part of the test in our study is 0.71.

### PTSD Checklist (PCL-5)

The PTSD Checklist (PCL-5) by Blevins et al., 2015 assesses the severity of the symptoms of Post-Traumatic Stress Disorder in adults [48]. We used the Polish adaptation of this scale according to the DSM-5 criteria by Ogińska-Bulik, et al., 2018 [42]. The tool comprises 20 items evaluating the severity of PTSD symptoms on a scale from 0 (not at all) to 4 (very strong). This scale has strong psychometric properties [48]. The Cronbach’s alpha coefficient in our study was 0.93.

### The State-Trait Anxiety Inventory (STAI)

The STAI questionnaire by CD Spielberger, et al., is designed to measure anxiety in adults. The instrument consists of two subscales, the first assesses anxiety as a transient situational state and the second as a relatively constant personality trait [49]. We used the STAI-T subscale (X-2) of the Polish adaptation of this questionnaire which rates trait anxiety [43]. The subscale consists of 20 items. Participants rate each item on a 5-point Likert scale ranging from 1 (not at all) to 5 (very much so) to indicate to what extent the behavior described in the question is typical for them [47]. This measure has demonstrated excellent psychometric properties [47]. Cronbach’s alpha coefficient in this study was 0.91.

### The Penn State Worry Questionnaire (PSWQ)

Penn State Worry Questionnaire by TJ Meyer, et al., 1990 is a 16-item inventory designed to assess pathological worry [50]. In the current study, we used the Polish adaptation by K. Janowski, 2007 [44]. Responses range from 1 (not at all typical of me) to 5 (very typical of me). This instrument has very strong psychometric properties [50]. The Cronbach’s alpha coefficient in our study was 0.94.

### Statistical analysis

To assess the distribution of analyzed variables, the Shapiro-Wilk test was carried out. Due to the non-Gaussian distribution of examined factors, non-parametric tests were used. The differences in examined factors were compared using the chi-square test for categorical variables, and a Mann-Whitney U-test for continuous variables. Comparisons were made between the following groups: 1) students who experienced at least one episode of SP in their life (SP +) and students who never experienced SP (SP-); 2) students who experienced 4 or more episodes of SP in a year and less than 4 episodes; 3) students who experienced 20 or more episodes of SP by the time of examination and less than 20 episodes; 4) reporting health problems and not reporting; 5) taking medications and not taking medications.

The relationship between observed variables was determined by using Spearman’s rho correlation. To evaluate the risk factors associated with sleep paralysis, we calculated the odds ratio (OR). Alpha Cronbach coefficient was calculated for reliability analysis of the psychological scales used in the study and subscale of SP-EPQ Questionnaire assessing psychosomatic symptoms of SP. A chi-square test was used to assess the differences between global prevalence and observed in the polish population, as same as differences between Irish and Polish populations’ prevalence of SP. For all analyses, p ≤ 0.05 was considered statistically significant. All analyses were carried out using Statistica software (TIBCO Software Inc., CA, USA).

## Results

### The demographic characteristics and health status of the study participants

The study participants (2553) were students of various universities in Poland. Among the students, the distribution of the represented fields of study was as follows: humanities 45.20% (*n* = 1154); technical 6.93% (*n* = 177); medical 23.85% (*n* = 609); natural science 20.13% (514); artistic 0.31%; economic 2.08% (*n* = 53); sports 0.78% (*n* = 20); military and marine 0.71% (*n* = 18). The detailed distribution of the represented fields of study among participants shows in Table S1.

The demographic characteristics and health status of the study participants are presented in Table 1.

**Table 1:** Participants’ demographic and health status characteristics by the group (*n* = 2553)

Group SP+/SP-:		SP +	SP -	p
**N**		846	1707	
**% Female (n)**		32.2 (656)	67.8 (1382)	0.04^a^
**% Male (n)**		36.9 (190)	63.1 (325)	
**Age**	*M* (*SD*)	22 (2.38)	21 (2.20)	
	Me	22.0	21.0	
	RNG	18–35	18–35	<0.001^a^
**Psychiatric disorder % (n)**	Anxiety	2.7% (23)	1.2% (20)	0.006^a^
	Mood	4.1% (35)	2.2% (37)	0.004^a^
	Others	–	0.5% (9)	
**Somatic disease % (n)**	Endocrine	7.3% (62)	6.3% (107)	0.13
	Allergic and Atopic	4.5% (38)	5.2% (88)	0.5
	Autoimmune	3.7% (31)	3.3% (57)	0.62
	Pulmonary	3.3% (28)	2.7% (46)	0.44
	Gastrointestinal	2.6% (22)	1.2% (21)	0.01^a^
	Cardiovascular	1.9% (16)	1% (17)	0.09
	Neurological	1.8% (15)	1.3% (22)	0.3
	Hematologic	1.2% (10)	0.5% (9)	0.05
**One health problem % (n)**		20.3% (172)	17.6% (300)	0.09
**Two health problem % (n)**		5.2% (44)	4.5% (76)	0.4
**Three or more health problem % (n)**		3.4% (29)	1.5% (26)	0.003^a^
**Medicines taken** **% (n)**	Antidepressants	6.3% (53)	2.5% (43)	0.02^a^
	Mood stabilizers	0.9% (8)	0.7% (12)	0.41
	Thyroid hormones	6.1% (52)	5.9% (100)	0.77
	Hormonal contraception	5.3% (45)	3.6% (62)	0.50
	Antiallergic and anti-asthmatic	3.9% (33)	4.5% (76)	0.51

*SP+* participants with at least one SP episode ever, *SP-* individuals who have not experienced SP, *SD* standard deviation, *M* mean, *Me* Median, *RNG* refers to range, *p* significance coefficients; ^a^ = statistically significant

### Point prevalence of SP

**Fig. 2:**
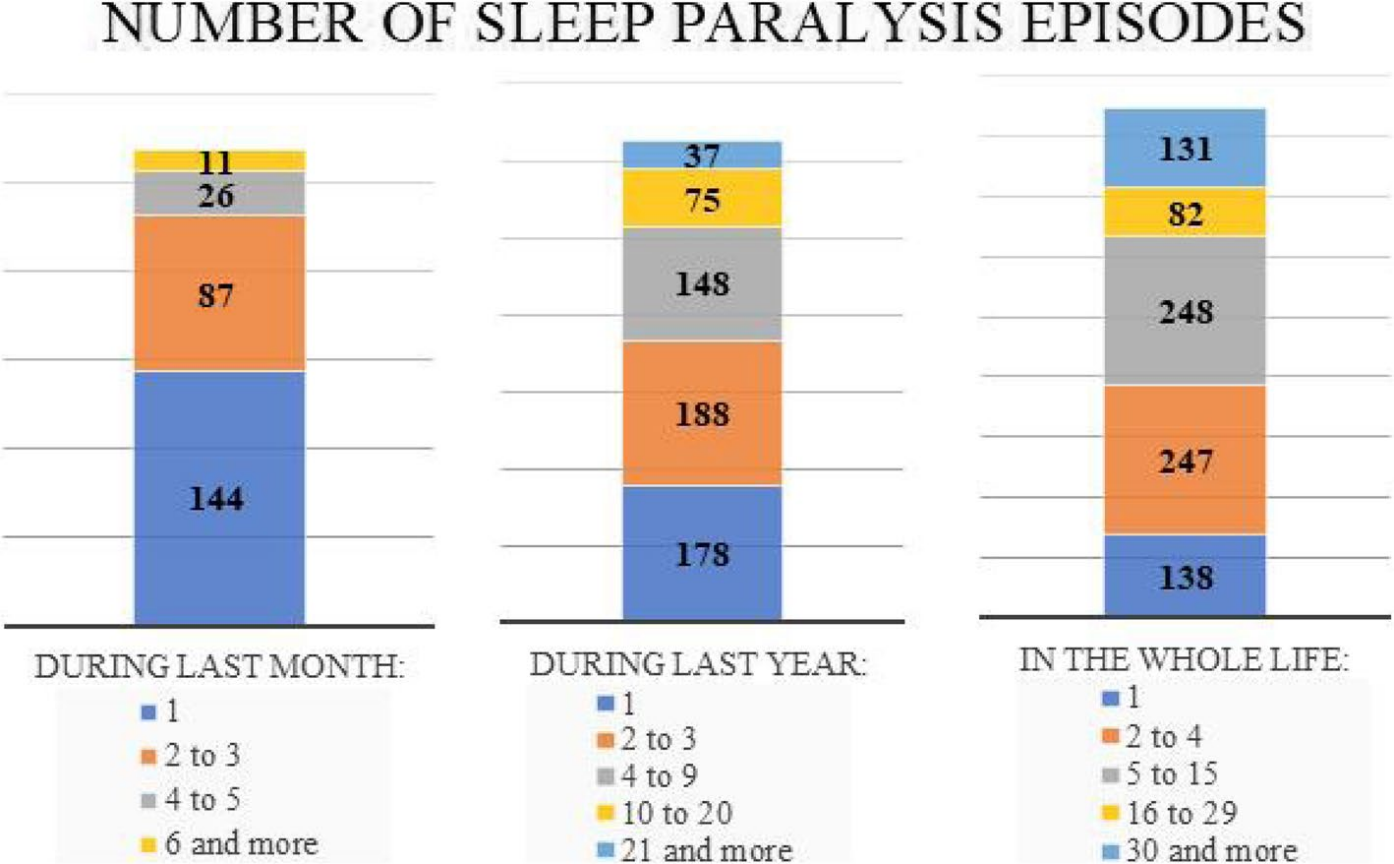
Histogram showing the distribution of sleep paralysis frequency-numbers of episodes during last month, last year, and whole life reported by participants of the study

Of the 2553 students participating in the study, 846 participants (33.14, 95%Cl: 31.31–34.96) experienced at least one SP episode ever (SP+) (32.2% F, 36.9% M). 260 students experienced 4 or more SP episodes in the last year (10.2%). The detailed frequency distribution of sleep paralysis episodes among participants who experienced at least 1 SP episode ever is shown in Fig. 2.

### Sleep paralysis and behavioral factors

#### Age

Participants who experienced at least 1 SP episode ever (SP+) were on average older than those who never experienced SP (SP-), M = 22.26, Me = 22.00, SD = 2.38 vs. M = 21.76, Me = 21.00, SD = 2.20 (Z = 5.62; p < 0.001).

#### Body Mass Index (BMI)

SP+ respondents had higher BMI compared to SP- participants, M = 22.83, Me = 04.22, SD = 4.50 vs. M = 22.47, Me = 21.55, SD = 5.77 (Z = 2.61; *p* = 0.009).

It was found that SP+ women had a significantly higher BMI than SP- women, M = 22.44, Me = 21.51, SD = 4.31 vs. M = 21.72, Me = 21.0, SD = 2.17 (Z = 2.61; *p* = 0.009)

#### Use of alcohol and cigarettes

In the smokers’ group, the odds ratio for SP was 1.48 higher than in non-smokers (OR: 1.48, 95% CI: 1.21–1.81, *p* = 0.0002).

SP+ subjects smoked more cigarettes per day and smoked longer compared to SP-participants, M = 1.64, Me = 0, SD = 4.0 vs. M = 1.24, Me = 0, SD = 3.59 (Z = 3.66; *p* = 0.0003); M = 1.12, Me = 0, SD = 2.40 vs. M = 0.81, Me = 0, SD = 2.25 (Z = 4.29; *p* = 0.00001).

SP+ women smoked more cigarettes and for a longer period of time compared to SP- women, M = 1.58, Me = 0, SD = 2.17 vs. M = 1.24, Me = 0, SD = 3.59 (Z = 3.66; *p* = 0.0003); M = 1.12, Me = 0, SD = 2.40 vs. M = 1.18, Me = 0, SD = 3.52 (Z = 3.66; *p* = 0.0003); The odds ratio for SP was 1.52 higher for subjects who reported alcohol consumption versus subjects reporting abstinence (OR: 1.52, 95% CI: 1.25–1.85, p < 0.0001).

It was observed that the SP+ subjects consumed alcohol significantly more often than the SP- subjects, M = 2.0 Me = 2.0 SD = 1.23 vs. M = 0.81, Me = 0, SD = 2.25 (Z = 4.29; *p* = 0.00001).

#### Sleep-related variables

The average sleep duration did not differ between SP+ and SP- participants.

Study participants who slept an average of 5 or fewer hours a day compared to participants who spent an average of 6 to 9.5 hours a day sleeping had more SP episodes in the last month, M = 1.09, Me = 0, SD = 1.64 vs. M = 0.68, Me = 0, SD = 1.50 (Z = 3.51; *p* = 0.0004) and year, M = 7.67, Me = 3, SD = 17.21 vs. M = 4.93, Me = 2, SD = 11.67 (Z = 2.68; *p* = 0.007).

Likewise women who slept an average of 5 or fewer hours compared to women who slept 6 to 9.5 hours a night had more SP episodes in the last month, M = 1.16, Me = 1, SD = 1.66 vs. M = 0.68, Me = 0, SD = 1.50 (Z = 3.51; *p* = 0.0004) and year, M = 7.67, Me = 3, SD = 17.21 vs. M = 4.51, Me = 2, SD = 9.86 (Z = 2.95; *p* = 0.003) and ever, M = 38.67, Me = 7, SD = 139.35 vs. M = 21.1, Me = 5, SD = 84.04 (Z = 2.28; *p* = 0.023).

In the group of men, there was no difference in the number of SP between those who slept less than 5 hours per day and those who slept from 6 to 9.5 hours per day.

Study participants who slept an average of 10 or more hours per day, compared with those who slept between 6 and 9.5 hours per day, had a higher incidence of SP episodes throughout their lives by the time of study, M = 22.04 Me = 10, SD = 27.91 vs. M = 23.34, Me = 5, SD = 106.15 (Z = 3.82; *p* = 0.0001).

In the group of studied women, sleep time was negatively correlated with the number of SP episodes in the last year (R = − 0.16; p < 0.008).

Among participants, 499 (58.98%) reported episodes of SP while sleeping on their back, 58 (6.86%) on the stomach, and 289 (34.16%) reported that the position did not matter. Sleep position (supine/prone) was not associated with increased frequency of SP.

#### Physical activity and SP prevalence

Playing sports was associated with a 1.31 higher odds ratio for developing SP versus lack of sports activity (OR: 1.31; 95% CI: 1.11–1.55; *p* = 0.001).

SP+ subjects spent more hours per week on physical activity compared to SP-, M = 1.99, Me = 1.0, SD = 1.2 vs. M = 1.63, Me = 0, SD = 2.26 (Z = 3.76; *p* = 0.0002). SP+ women spent more hours during the week engaged in physical activity compared to SP- women, M = 1.75, Me = 0, SD = 2.22 vs. M = 1.63, Me = 0, SD = 2.26 (Z = 3.76; *p* = 0.0002).

Participants who experienced twenty or more episodes of SP by the time of the study spent more hours per week on physical activity compared to those who had fewer episodes or never experienced SP, M = 2.22, Me = 1.0, SD = 2.50 vs. M = 1.70, Me = 0, SD = 2.31 (Z = 3.44; *p* = 0.0006).

There was no correlation between the number of hours spent on physical activity and the number of SP episodes (p < 0.05). There were also no differences in the frequency of SP between the groups that were devoted to sports: > = 10 h / 5–9 h / <= 4 h per week (p < 0.05).

#### The relationship between the frequency of SP and the occurrence of other diseases and drug use

In this section, only students who have had at least one episode of SP in their lifetimes were included. These students were divided into groups depending on:

I. Presence of psychiatric disorder- 1) students who reported none and 2) students who reported at least one mental disorder (any/mood/anxiety).
II. Presence of somatic disease- 1) students physically healthy and 2) students who did report somatic disease (allergies/asthma/ cardiovascular/hematologic/gastrointestinal).
III. The number of health problems reported (mental or somatic)- 1) without any health problems 2) one or 3) two or 4) three or more health problems.
IV. Medication taken- 1) students who reported no medications taken and 2) those who did report taking prescription medications (any medications/antidepressants/antiallergic and anti-asthmatic / hormonal contraception/thyroid hormones).

### The prevalence of SP and the presence of other psychiatric disorders

The subjects who reported any mental disorders had more SP episodes during their lifetime compared to healthy subjects, M = 23.70, Me = 10, SD = 45.3 vs. M = 25.77, Me = 5, SD = 116.44 (Z = 2.62; *p* = 0.009). The presence of any type of mental disorder in the subjects was associated with a 1.77 higher odds ratio for SP compared to the mentally healthy subjects (OR: 1.77; 95% CI: 1.22–2.55; *p* = 0.002).

In the SP+ group, people who reported mental disorders (*n* = 56) were older, had a higher BMI, smoked more cigarettes and for a longer period, had more PTSD symptoms reflected by the PCL-5 scale, and had more SP episodes throughout their lives compared to mentally healthy people, see Table 2.

**Table 2:** Participants who experienced at least one SP episode in their lives - differences between those who reported mental disorders and those who did not report them

	SP+									
	With psychiatric disorders				Without psychiatric disorders					
	Me	M	Range	IQR	Me	M	Range	IQR	Z	p
**Age**	23.0	22.95	15.0	3.00	22	22.21	16.0	3.00	2.22	0.026
**BMI**	23.01	24.42	17.31	4.27	21.97	22.71	47.45	4.96	2.09	.036
**Number of cigarettes smoked**	0	3.05	1.0	0.25	0	1.54	1.0	0	2.78	0.005
**Number of pack-year**	0	2.25	11.25	0.60	0	1.04	10.0	0	3.27	0.001
**PCL-5**	33.5	32.66	65.0	26.0	28.0	28.33	72.0	26.0	2.04	0.041
**The number of SP episodes throughout the lifetime**	10.0	23.34	299.0	16.0	5.0	25.79	1999.0	13.0	2.56	0.01

*SP+* participants with at least one lifetime episode of SP ever, *Me* median, *M* mean, *IQR* interquartile range, *Z* score, *p* significance coefficients

Study participants who reported a mood disorder had a 2.07-fold higher risk of developing SP compared to patients who did not have a mood disorder (OR: 2.07; 95% CI 1.29–3.31; *p* = 0.002).

Significant correlations between the frequency of SP episodes and lifestyle variables were found among participants reporting: any mental disorders, anxiety disorders, and mood disorders, see Table 3.

**Table 3:** Correlation between age, BMI, lifestyle variables, and SP frequency during the last month, year, and lifetime in individual groups of participants

**The number of SP episodes:**	**Participants reported mental disorder**			**Participants reported somatic disease**				**Participants reported two or more health problem**	
	**Any mental disorders**	**Anxiety**	**Mood**	**Allergies**	**Asthma**	**Cardiovascular**	**Hematologic**	**Two health problem**	**Three or more health problem**
**in the last month**	-frequency of alcohol consumption per month, R = 0.28 - Average sleep duration in hours per day, R = −0.32				-number of coffee cups per day, R = 0.5		-BMI, R = 0.7	number of coffee cups per day, R = 0.31	
**in the last year**	- number of hours of physical activity per week, R = 0.47	- average sleep duration in hours per day, R = −0.51	- number of hours of physical activity per week, R = 0.51	-age, R = −0.33	-number of coffee cups per day, R = 0.49 - average sleep duration in hours per day, R = −0.42	-age, R = −0.63	- average sleep duration in hours per day, R = −0.72		-age, R = −0.42
**throughout the lifetime**	- number of hours of physical activity per week, R = 0.43	- average sleep duration in hours per day, R = −0.47 - number of hours of physical activity per week, R = 0.43	- number of hours of physical activity per week, R = 0.48					-number of coffee cups per day, R = 0.4 -number of hours of physical activity per week, R = 0.38	
	**Participants reporting regularly taking medication**								
**Number of SP episodes:**	**Any medications**		**thyroid hormones**		**hormonal contraception**		**antidepressants**	**antiallergic and anti-asthmatic**	
**in the last month**	- average sleep duration in hours per day, R = −0.33								
**in the last year**	- number of hours of physical activity per week, R = 0.46				-BMI, R = 0.34 -number of coffee cups per day, R = 0.36		-average sleep duration in hours per day, R = 0.39	-age, R = 0.41	
**throughout the lifetime**	- number of hours of physical activity per week, R = 0.35		- number of cigarettes smoked per day, R = 0.34 - number of pack-years, R = 0.34				- average sleep duration in hours per day, R = 0.36	-age, R = 0.42	

The correlation between age, BMI, lifestyle variables, and the number of SP episodes during the last month, year, and lifetime in individual groups of participants using the Spearman’s rank correlation coefficient (R). Significance coefficients, *p* < 0.05

### The prevalence of SP and the presence of other somatic disease

The subjects who reported any type of somatic disease had more SP episodes during their lifetime compared to healthy subjects, M = 21.74, Me = 6, SD = 70.75, vs. M = 27.24, Me = 5, SD = 126,58 (Z = 2,78; *p* = 0,005). The presence of any type of somatic disorder was associated with a 1.34-fold higher odds ratio of developing SP (OR: 1.34; 95% CI 1.11–1.61; *p* = 0.002).

In the group of patients with three health problems, the odds ratio for developing SP was 2.3 times higher than in those who had fewer health problems or were healthy (OR: 2.3; 95% CI 1.35–3.93; *p* = 0.002).

Students who reported hematological disorder had a 2.54 times higher odds ratio for SP compared to healthy people (OR: 2.54; 95% CI 1.00–6.47; *p* = 0.05).

Individuals with gastrointestinal disorders had a 2.26-fold higher odds ratio for SP development compared with persons without gastrointestinal disease (OR: 2.26; 95% CI 1.22–4.16; *p* = 0.009).

Responders with cardiovascular disease had a 2.15-fold higher odds ratio for SP development compared with persons without cardiovascular disease (OR: 2.15; 95% CI 1.17–3.93; *p* = 0.01).

Significant correlations between the frequency of SP episodes and age, BMI, and lifestyle variables were found among participants reporting: allergies, asthma, cardiological diseases, haematological disorder, two health problem, and three or more health problems, see Table 3.

### The prevalence of SP and drug intake

Patients taking antidepressants had a 1.58 higher odds ratio for developing SP compared to those not taking antidepressants (OR: 1.58; 95% CI 1.09–2.29; *p* = 0.01).

Significant correlations between the frequency of SP episodes and age, BMI, and lifestyle variables were found among participants reporting taking medications regularly: any medications, thyroid hormones, hormonal contraception, antidepressants, antiallergic and anti-asthmatic, see Table 3.

### The relationship between SP and the severity of anxiety symptoms

#### SP and symptoms of anxiety as a personality trait

A higher level of anxiety as a constant personality trait (number of points in the STAI questionnaire> = 44) was associated with a 1.20 higher odds ratio for SP (OR: 1.20, 95% CI: 1.01–1.41; *p* = 0.035).

The SP+ participants had a significantly higher level of anxiety understood as a personality trait compared to the SP- participants, M = 47.9, Me = 48.0, SD = 10.74 M vs. M = 46.9, Me = 46.0, SD = 10.77 (Z = 2.12; *p* = 0.034).

Among SP+ men, the severity of symptoms measured with the STAI questionnaire was higher than among SP- men, M = 48.1, Me = 48.0, SD = 11.18 vs. M = 43.8, Me = 44.0, SD = 10.47 (Z = 4.09; *p* = 0.00004).

The severity of anxiety symptoms did not differ between SP+ women and SP- women (*p* > 0.05).

Participants who experienced twenty or more episodes of SP by the time of the study had higher STAI scores compared to those who had fewer episodes or never experienced SP, M = 48.67, Me = 49.5, SD = 10.59 vs. M = 47.12, Me = 47.0, SD = 10.78 (Z = 2.12; *p* = 0.034).

#### SP and a tendency to worry

It was found that SP+ men had a higher tendency to worry (they had a higher score on the PSWQ) compared to SP- men, M = 52.83, Me = 53.0 SD = 13.96 vs. M = 47.69, Me = 48.0, SD = 14.73 (Z = 3.71; *p* = 0.0002).

#### SP and severity of symptoms of Post-Traumatic Stress Disorder (PTSD)

The severity of PTSD symptoms did not differ between those with SP and those who never experienced SP (*p* > 0.05).

## Discussion

The aim of our study was to assess the prevalence of SP and to identify potential factors which could be predictors of its in a Polish student population. Sleep paralysis remains a poorly researched disorder and work is underway to clarify the risk factors for its occurrence.

The point prevalence of SP in the population of Polish students was 33% which does not significantly differ statistically from the average prevalence in students worldwide, estimated at 28.3% (chi2 = 0,104). To the best of our knowledge, among other European countries, only Ireland has carried out a study on a student population, with a prevalence rate of SP - 19.9% [51], which is also not statistically significantly lower than in our country (chi2 = 0,5) [14]. The highest prevalence in the student population was recorded in Peru namely 55% [52]. In student populations within other countries, the prevalence of SP varies by study, e.g. in Canada, 29 to 41.9% of students have experienced at least one episode of SP [53, 54]; in the USA about 25% [55], in Japan from 38.9 to 43% [54, 56]; in Nigeria, from 26.2 to 44.2% [57, 58]; in Egypt 43% [10], in Kuwait about 29% and in Sudan 30% [59].

Our assumptions regarding the association of SP with lifestyle factors have been partially supported. We found an association between SP and BMI, alcohol consumption, smoking, sleep duration, and physical activity. Subjects who experienced at least 1 SP ever had a higher BMI compared to those who had never experienced it. Similar results were obtained by Sharpless et al., 2010, which showed a positive correlation between the number of FISP (Fearful Isolated Sleep Paralysis) and the BMI of the subjects [8]. It is interesting that in a 2017 study on the student population by Abdel Wahed and Hassan, BMI over 25 was associated with higher levels of anxiety and stress [60]. The relationship between the incidence of SP and the use of psychoactive substances such as alcohol and cigarettes is not surprising. Previous studies have already supported this relationship [19, 24]. Munezawa et al., in their study in a group of 90,081 Japanese teenagers, found a higher incidence of SP among alcohol users (7.1% vs 12.2%) and cigarette smokers (7.8% vs 15.3%) compared to those who did not use these substances [19]. It is interesting, however, that in this population of Polish students, the use of these psychoactive substances is associated with a higher odds ratio for SP by as much as 1.52 for alcohol and 1.48 for smoking.

Our observations supported that the incidence of SP may be related to the duration of sleep [24, 31, 61]. The mean time of sleep per day was negatively correlated with the number of SP episodes during the last year in women. There was a negative correlation in participants who suffered from hematological disorders, asthma, and anxiety disorders. In addition, study participants who slept an average of 5 or fewer hours a night compared to participants who spent an average of 6 to 9.5 hours a night sleeping had more SP during the last month and year. Furthermore, too much sleep (> = 10 h) was associated with a higher number of SP episodes throughout a lifetime. Munezawa et al., 2011 made similar observations in a group of Japanese teenagers studied, wherein the incidence of SP was higher in those who slept less than 5 hours or more than 9 hours per day [19].

The results we obtained regarding the influence of physical activity on the incidence of SP may seem controversial in light of many studies that document the beneficial effect of physical activity on sleep quality [62, 63]. However, it is important to remember that the effect of exercise on sleep depends on gender, age, BMI, condition, type, and protocol of exercise (intensity, duration, and type of exercise) [63, 64]. Vigorous exercise can significantly affect sleep architecture, increasing the delay in slow wave (SWS) and paradoxical (REM) sleep, reducing the amount of REM sleep [65, 66]. In our study, we did not assess the usual hours of physical exercise and sleep, and as the research suggests, it may be related to the occurrence of sleep disorders, because a negative impact on sleep latency and awakening at night was noticed in people who exercise earlier than 8 hours or less than 4 hours before bedtime [65, 66]. A further investigation extending the research methods with a detailed assessment of the exercise schedule and normal hours of sleep appears worthy of pursuit.

Results of research by Ruotolo et al., 2016, showed that children involved in physical activity suffered from parasomnia more often (62%) than physically inactive children (38%) which should be noted [67]. To date, the effect of physical activity on the incidence of SP has not been explored. Our results indicate that there was higher physical activity in participants who experienced at least one episode of SP compared to those who never experienced the phenomenon. Additionally, there is a positive correlation between the number of hours spent on physical activity and the number of SP episodes in people with at least two health problems, mental disorders, or those taking antidepressants which suggests that exercise may be related to SP in some extent. The subject requires further study, bearing in mind that the level of physical activity was not tested by us with special questionnaires, but only with a proprietary sociodemographic survey, which included two questions about sport, namely: “Do you do sports?” and “If so, how many hours a week do you do physical activity?”

As we showed in our study, people with an additional burden of somatic disease have a higher risk of developing SP and more often experience episodes of SP. Little research has been done to date on the relationship between SP and other diseases. Mume and Ikem, 2009 showed that there are significant differences in the prevalence of SP between healthy and sick people. In their study, SP was reported by 28% of healthy people, 44% of orthopedic patients, and 56% of patients complaining of multiple somatic complaints [37]. In a study by Takeuchi et al., 2002, the group of subjects who experienced isolated SP exhibited poorer performance, more complaints of physical, mental, and neurotic symptoms, and increased subjective fatigue compared to subjects who never experienced SP [36]. Moreover, SP occurrences were more frequent with obstructive sleep apnea and arterial hypertension [33, 36].

The correlation between the occurrence of mental disorders and more frequent SP episodes has been confirmed in many studies on sleep paralysis [7, 14, 68]. The results of our study reveal that a high level of anxiety understood as a personality trait (> 44 points) is associated with as much as a 1.20 higher odds ratio for SP. Moreover, participants who experienced at least one episode of SP in their lifetime had significantly higher symptoms of anxiety, which is understood as a personality trait, compared to those who never experienced it. Interestingly, this relationship was observed especially in men. There were no significant differences in anxiety levels among women. In men who experienced at least 1 SP episode in their lifetime, the severity of worry was significantly higher compared to those who had never experienced it. The results of our research suggest that high levels of anxiety as a personality trait and a tendency to worry may be risk factors for developing SP. Jalal and Hinton (2015) also observed such a relationship in the Egyptian student population [10]. The relationship between the presence of anxiety symptoms is also confirmed by the observations of other researchers [13, 17, 26, 34, 69]. Importantly, our research shows that people suffering from mental disorders were not only at significant risk of developing SP but also had significantly more episodes of SP throughout their lives compared to people with a negative history of mental illness. In the SP+ group, people suffering from mental disorders (*n* = 56) were older, had a higher BMI, smoked more cigarettes for a longer period, had more PTSD symptoms, and had more incidences of SP throughout their lives compared to mentally healthy people, see Table 2. Interestingly, the results of our study indicate that taking antidepressants is associated with an increased risk of SP. This is surprising, considering that other studies have not supported the effect of these drugs on the incidence of SP [8, 18, 21]. Moreover, there are reports of successful treatment of SP with SSRIs and TLPDs [70–72]. On the other hand, it is a fact that some classes of antidepressants may worsen the quality of sleep due to the activation of 5-HT2 serotonergic receptors and increased noradrenergic and dopaminergic neurotransmission. In addition, some drugs from the SSRI, SNRI, and TCA groups also have a negative impact on sleep documented by research [73]. They induce or worsen bruxism during sleep, may induce nightmares, and disrupt muscle tone during REM sleep, which may induce or exacerbate the movement disorder associated with the REM sleep phase [73]. The effect of antidepressants depends on both the class and their dose, so it would be important to carefully study the effect of these drugs on SP. It can be assumed that the more frequent occurrence of SP may be conditioned by many factors, as a component of lifestyle, mental and physical health.

In our study, we did not support an association of SP with PTSD which was found in other studies. However, this can be explained by the fact that the student population, despite being exposed to chronic stress, is not at risk of Post-Traumatic Stress Syndrome [38, 74].

The study has several limitations. The first is the form of an online questionnaire which makes it impossible to clarify the question if the researcher feels it is unclear. The second is Sampling Bias, namely that people suffering from SP could more likely to respond to the survey than people who did not experience this phenomenon, which might have influenced the result of the prevalence of SP in the Polish student population. Large sample size may influence the results of a study by showing significant statistical differences even when they are clinically insignificant. Another is that the number of SP risk factors in our online survey was limited. We assume that there are other risk factors influencing the prevalence of SP in the Polish student population. In the future, it would be interesting to extend the research methods to include assessment tools: stress exposure, ways of coping with stress, assessment of depressive symptoms, hygiene and quality of sleep, sleep disorders, physical activity; taking into account the frequency, intensity, and type of training of participants, as well as the usual time of exercise. Another limitation of the study is that women accounted for 80% of the respondents. The reason for this may be that in Poland, in 2018, women dominated at most Universities [75]. Women accounted for 70% of students of humanities and medicine, and almost 70% of those studying art and natural sciences [75]. In the future, it would be worthwhile to conduct a study of faculties focusing on male students. Further research is required to establish SP risk factors in the Polish population.

The results of our research indicate that a large proportion of students experienced isolated sleep paralysis. The variables predisposing to this disorder include both psychological conditions (tendency to worry, high level of anxiety as a constant personality trait), general health (the presence of somatic and mental disorders) as well as behavioral factors (psychoactive substances, sleep time, level of physical activity, medicines). Considering the large scale of the problem, detailed studies would be warranted to further investigate the influence of these variables on the frequency and course of sleep paralysis. Accurate determination of predictors is essential to designing preventive and therapeutic interventions.

## Conclusions

1. The point prevalence of SP in the population of Polish students was 33% which does not differ statistically significantly from the average worldwide prevalence in students, estimated at 28.3%.

2. Smoking, alcohol consumption, physical activity, the presence of somatic and mental disorders, taking antidepressants, and higher level of anxiety as constant personality trait are associated with an increased odds ratio for SP in the Polish student population.

3. Older age, higher BMI, and sleep duration (too short [<= 5 h / d] or too long [> = 10 h / d]) were associated with a more frequent occurrence of SP in our study population.

4. Individuals burdened with other somatic and mental disorders have more SP compared to healthy people.

5. Further research is recommended to determine the mechanisms by which these factors influence the course of development of SP.

## Supplementary Information

**Additional file 1: Table S1**. Fields of study among participants.

## Data Availability

The datasets analyzed during the current study are not publicly available but are available from the corresponding author on reasonable request.

## Abbreviations

SP: Sleep paralysis
REM: Rapid eye movement sleep
SP-EPQ: Sleep Paralysis Experience and Phenomenology Questionnaire
PCL -5: PTSD Checklist
STAI-T: The State-Trait Anxiety Inventory
PSWQ: The Penn State Worry Questionnaire
ISP: Isolated Sleep Paralysis
FISP: Fearful Isolated Sleep Paralysis
PTSD: Post-Traumatic Stress Disorder
GAD: Generalized Anxiety Disorder
BMI: Body Mass Index
SPQ: Sleep Paralysis Questionnaire
OR: Odds ratio
F: Female
M: Male
SWS: Slow-wave sleep

## References

[1] Sharpless BA (2016). A clinician’s guide to recurrent isolated sleep paralysis. Neuropsychiatr Dis Treat.

[2] Raduga M. Optimal Sleep Duration and Its Deviation Outcomes From Perspectives of REM Sleep Dissociative Phenomena. Dreaming. 2021;31:244.

[3] Sharpless BA, Grom JL (2016). Isolated Sleep Paralysis: Fear, Prevention, and Disruption. Behav Sleep Med.

[4] Sharpless BA, Kliková M (2019). Clinical features of isolated sleep paralysis. Sleep Med.

[5] Cheyne JA, Girard TA (2009). The body unbound: Vestibular-motor hallucinations and out-of-body experiences. Cortex..

[6] Ropper AH, Samuels MA, Klein JP (2014). Chapter 19. Sleep and Its Abnormalities. Adams and Victor’s Principles of Neurology, 10e.

[7] Denis D, French CC, Gregory AM (2018). A systematic review of variables associated with sleep paralysis. Sleep Med Rev.

[8] Sharpless BA, McCarthy KS, Chambless DL, Milrod BL, Khalsa SR, Barber JP (2010). Isolated sleep paralysis and fearful isolated sleep paralysis in outpatients with panic attacksb. J Clin Psychol.

[9] Jalal B (2018). The neuropharmacology of sleep paralysis hallucinations: serotonin 2A activation and a novel therapeutic drug. Psychopharmacology.

[10] Jalal B, Hinton DE (2015). Sleep paralysis among egyptian college students: Association with anxiety symptoms (PTSD, trait anxiety, pathological worry). J Nerv Ment Dis.

[11] American Academy of Sleep Medicine. ICSD-3 Online Version - American Academy of Sleep Medicine (AASM). Darien: AASM; 2014.

[12] Denis D (2018). Relationships between sleep paralysis and sleep quality: Current insights. Nat Sci Sleep.

[13] Denis D, French CC, Rowe R, Zavos HMS, Nolan PM, Parsons MJ (2015). A twin and molecular genetics study of sleep paralysis and associated factors. J Sleep Res.

[14] Sharpless BA, Barber JP (2011). Lifetime prevalence rates of sleep paralysis: A systematic review. Sleep Med Rev.

[15] Hsieh SW, Lai CL, Liu CK, Lan SH, Hsu CY (2010). Isolated sleep paralysis linked to impaired nocturnal sleep quality and health-related quality of life in Chinese-Taiwanese patients with obstructive sleep apnea. Qual Life Res.

[16] Ramsawh HJ, Raffa SD, White KS, Barlow DH (2008). Risk Factors for Isolated Sleep Paralysis in an African American Sample: A Preliminary Study. Behav Ther.

[17] Spanos NP, McNulty SA, DuBreuil SC, Pires M, Burgess MF (1995). The frequency and correlates of sleep paralysis in a university sample. J Res Pers.

[18] Otto MW, Simon NM, Powers M, Hinton D, Zalta AK, Pollack MH (2006). Rates of isolated sleep paralysis in outpatients with anxiety disorders. J Anxiety Disord..

[19] Munezawa T, Kaneita Y, Osaki Y, Kanda H, Ohtsu T, Suzuki H (2011). Nightmare and sleep paralysis among Japanese adolescents: A nationwide representative survey. Sleep Med.

[20] Wing YK, Lee ST, Chen CN (1994). Sleep paralysis in Chinese: Ghost oppression phenomenon in Hong Kong. Sleep..

[21] Ohayon MM, Zulley J, Guilleminault C, Smirne S (1999). Prevalence and pathologic associations of sleep paralysis in the general population. Neurology..

[22] Young E, Xiong S, Finn L, Young T (2013). Unique sleep disorders profile of a population-based sample of 747 Hmong immigrants in Wisconsin. Soc Sci Med.

[23] Jalal B, Simons-Rudolph J, Jalal B, Hinton DE (2014). Explanations of sleep paralysis among Egyptian college students and the general population in Egypt and Denmark. Transcult Psychiatry.

[24] Ma S, Wu T, Pi G (2014). Sleep paralysis in Chinese adolescents: A representative survey. Sleep Biol Rhythms.

[25] Lišková M, Janečková D, Klůzová Kráčmarová L, Mladá K, Bušková J (2016). The occurrence and predictive factors of sleep paralysis in university students. Neuropsychiatr Dis Treat.

[26] Hinton DE, Pich V, Chhean D, Pollack MH, McNally RJ (2005). Sleep paralysis among Cambodian refugees: Association with PTSD diagnosis and severity. Depress Anxiety.

[27] Mellman TA, Aigbogun N, Graves RE, Lawson WB, Alim TN (2008). Sleep paralysis and trauma, psychiatric symptoms and disorders in an adult African American population attending primary medical care. Depress Anxiety..

[28] Abrams MP, Mulligan AD, Carleton RN, Asmundson GJG (2008). Prevalence and correlates of sleep paralysis in adults reporting childhood sexual abuse. J Anxiety Disord..

[29] McNally RJ, Clancy SA (2005). Sleep paralysis in adults reporting repressed, recovered, or continuous memories of childhood sexual abuse. J Anxiety Disord.

[30] Denis D, French CC, Schneider MN, Gregory AM. Subjective sleep-related variables in those who have and have not experienced sleep paralysis. J Sleep Res. 2018;27:e12650. 10.1111/jsr.12650 29280229

[31] Munezawa T, Kaneita Y, Yokoyama E, Suzuki H, Ohida T (2009). Epidemiological study of nightmare and sleep paralysis among Japanese adolescents. Sleep Biol Rhythms.

[32] Dodet P, Chavez M, Leu-Semenescu S, Golmard J-L, Arnulf I (2015). Lucid Dreaming in Narcolepsy. Sleep..

[33] Vernet C, Redolfi S, Attali V, Konofal E, Brion A, Frija-Orvoen E (2011). Residual sleepiness in obstructive sleep apnoea: Phenotype and related symptoms. Eur Respir J.

[34] Denis D, Poerio GL (2017). Terror and bliss? Commonalities and distinctions between sleep paralysis, lucid dreaming, and their associations with waking life experiences. J Sleep Res.

[35] Sharpless BA (2015). Exploding head syndrome is common in college students. J Sleep Res.

[36] Takeuchi T, Fukuda K, Sasaki Y, Inugami M, Murphy TI (2002). Factors related to the occurrence of isolated sleep paralysis elicited during a multi-phasic sleep-wake schedule. Sleep..

[37] Mume CO, Ikem IC (2009). Sleep paralysis and psychopathology. South African J Psychiatry..

[38] Grochmal-bach BI (1996). Problemy dezadaptacji studentów i próby oddziaływań pedagogiczno-terapeutycznych.

[39] Romanowska-Tołłoczko A (2011). Styl życia studentów oceniany w kontekście zachowań zdrowotnych. Hygeia Public Heal.

[40] Wróbel-Knybel P, Karakuła-Juchnowicz H, Flis M, Rog J, Hinton DE, Boguta P (2020). Prevalence and clinical picture of sleep paralysis in a Polish student sample. Int J Environ Res Public Health.

[41] Jalal B, Hinton DE (2013). Rates and Characteristics of Sleep Paralysis in the General Population of Denmark and Egypt. Cult Med Psychiatry.

[42] Ogińska-Bulik N, Juczyński Z, Lis-Turlejska MM-KD (2018). Polska adaptacja PTSD Check List for DSM-5 – PCL-5. Doniesienie wstępne.

[43] Wrześniewski K, Sosnowski T, Jaworowska A, Fecenec D. STAI – Inwentarz Stanu i Cechy Lęku. Polska adaptacja STAI. Warszawa: Pracownia Testów Psychologicznych PTP; 2011.

[44] Meyer TJ, Miller ML, Metzger RL, Borkovec TD. Development and validation of the Penn State Janowski K. Kwestionariusz Oceny Martwienia się, polska adaptacja PSWQ. Katedra Psychologii Klinicznej. Lublin: KUL Behav Res Ther. 2007;28:487–95. 10.1016/0005-7967(90)90135-6 2076086

[45] Wróbel-Knybel P, Rog J, Jalal B, Szewczyk P, Karakuła-Juchnowicz H (2021). Sleep paralysis among professional firefighters and a possible association with PTSD—Online survey-based study. Int J Environ Res Public Health.

[46] Jalal B, Romanelli A, Hinton DE (2020). Sleep paralysis in Italy: Frequency, hallucinatory experiences, and other features. Transcult Psychiatry..

[47] Jalal B, Sevde Eskici H, Acarturk C, Hinton DE (2020). Beliefs about sleep paralysis in Turkey: Karabasan attack. Transcult Psychiatry..

[48] Blevins CA, Weathers FW, Davis MT, Witte TK, Domino JL (2015). The Posttraumatic Stress Disorder Checklist for DSM-5 (PCL-5): Development and Initial Psychometric Evaluation. J Trauma Stress.

[49] Gaudry E, Vagg P, Spielberger CD (1975). Validation of the state-trait distinction in anxiety research. Multivariate Behav Res.

[50] Meyer TJ, Miller ML, Metzger RL, Borkovec TD (1990). Development and validation of the penn state worry questionnaire. Behav Res Ther.

[51] O’Hanlon J, Murphy M, Di Blasi Z (2011). Experiences of sleep paralysis in a sample of Irish university students. Ir J Med Sci.

[52] Huamaní C, Martínez A, Martínez C, Reyes A (2013). Prevalencia y presentación de la parálisis del sueño en estudiantes de Medicina Humana de la UNMSM. An la Fac Med.

[53] Cheyne JA, Rueffer SD, Newby-Clark IR (1999). Hypnagogic and Hypnopompic Hallucinations during Sleep Paralysis: Neurological and Cultural Construction of the Night-Mare. Conscious Cogn.

[54] Fukuda K, Ogilvie RD, Chilcott L, Vendittelli AM, Takeuchi T (1998). The prevalence of sleep paralysis among Canadian and Japanese college students. Dreaming..

[55] Paradis C, Friedman S, Hinton DE, McNally RJ, Solomon LZ, Lyons KA (2009). The assessment of the phenomenology of sleep paralysis: The unusual sleep experiences questionnaire (USEQ). CNS Neurosci Ther.

[56] Fukuda K, Miyasita A, Inugami M, Ishihara K (1987). High prevalence of isolated sleep paralysis: kanashibari phenomenon in Japan. Sleep..

[57] Ohaeri JU, Adelekan MF, Odejide AO, Ikuesan BA (1992). The pattern of isolated sleep paralysis among Nigerian nursing students. J Natl Med Assoc.

[58] Ohaeri JU, Odejide AO, Ikuesan BA, Adeyemi JD (1989). The pattern of isolated sleep paralysis among Nigerian medical students. J Natl Med Assoc.

[59] Awadalla A, Amfayez G, Harville M, Arikawa H, Tomeo ME, Templer DI (2004). Comparative prevalence of isolated sleep paralysis in Kuwaiti, Sudanese, and American college students. Psychol Rep.

[60] Abdel Wahed WY, Hassan SK (2017). Prevalence and associated factors of stress, anxiety and depression among medical Fayoum University students. Alexandria J Med.

[61] Jalal B, Ramachandran VS (2014). Sleep paralysis and ‘the bedroom intruder’: The role of the right superior parietal, phantom pain and body image projection. Med Hypotheses.

[62] Farnsworth JL, Kim Y, Kang M. Sleep disorders, physical activity, and sedentary behavior among U.S. adults: National health and nutrition examination survey. J Phys Act Heal. 2015;12:1567–75. 10.1123/jpah.2014-0251 25710522

[63] Chennaoui M, Gomez-Merino D, Arnal P, Sauvet F, Léger D. Is there an interrelationship between sleep and exercise? Med du Sommeil. 2015;12(4):169–80.

[64] Chen YS, Chen MC, Chou FHC, Sun FC, Chen PC, Tsai KY, et al. The relationship between quality of life and posttraumatic stress disorder or major depression for firefighters in Kaohsiung, Taiwan. Qual Life Res. 2007;16(8):1289–97. 10.1007/s11136-007-9248-7 17668289

[65] Kubitz KA, Landers DM, Petruzzello SJ, Han M (1996). The Effects of Acute and Chronic Exercise on Sleep A Meta-Analytic Review. Sports Med.

[66] Youngstedt SD, O’Connor PJ, Dishman RK (1997). The effects of acute exercise on sleep: A quantitative synthesis. Sleep..

[67] Ruotolo F, Prado LBF, Ferreira VR, Prado GF, Carvalho LBC (2015). Intake of stimulant foods is associated with development of parasomnias in children. Arq Neuropsiquiatr.

[68] Yeung A, Xu Y, Chang DF (2005). Prevalence and Illness Beliefs of Sleep Paralysis among Chinese Psychiatric Patients in China and the United States. Transcult Psychiatry..

[69] Bell CC, Hildreth CJ, Jenkins EJ, Carter C (1988). The relationship of isolated sleep paralysis and panic disorder to hypertension. J Natl Med Assoc.

[70] Koran LM, Raghavan S (1993). Fluoxetine for Isolated Sleep Paralysis. Psychosomatics..

[71] Hishikawa Y, Ida H, Nakai K, Kaneko Z (1966). Treatment of narcolepsy with imipramine (Tofranil) and desmethylimipramine (Pertofran). J Neurol Sci.

[72] Stores G (2003). Medication for sleep-wake disorders. Arch Dis Child..

[73] Wichniak A, Wierzbicka A, Walęcka M, Jernajczyk W (2017). Effects of Antidepressants on Sleep. Curr Psychiatry Rep.

[74] Tortella-Feliu M, Fullana MA, Pérez-Vigil A, Torres X, Chamorro J, Littarelli SA (2019). Risk factors for posttraumatic stress disorder: An umbrella review of systematic reviews and meta-analyses. Neurosci Biobehav Rev.

[75] Nauki M, Wy S. Szkolnictwo wyższe w Polsce w latach 2012–2018. Ministerswto Edukacji i Nauki 2019. Warszawa; 2019. https://radon.nauka.gov.pl.

