# Supplementary material for: Risk factors of sleep paralysis in a population of Polish students
Source: BMC Psychiatry. 2022 Jun 7;22:383. doi: 10.1186/s12888-022-04003-0 (PMC9171979; doi:10.1186/s12888-022-04003-0)
Supplement: Supplementary file 1 — Additional file 1: Table S1. Fields of study among participants. [file 12888_2022_4003_MOESM1_ESM.docx]

| *Table S1. Fields of study among participants.* | | | |
| --- | --- | --- | --- |
| **Fields of study:** | **% Student (n)** | **% Female (n)** | **% Male (n)** |
| **Humanistic** | 45.20 (1154) | 82.24 (949) | 17.76 (205) |
| **Technical** | 6.93 (177) | 55.37 (98) | 44.63 (79) |
| **Medical** | 23.85 (609) | 84.40 (496) | 18.60 (113) |
| **Natural Science** | 20.13 (514) | 81.79 (420) | 18.29 (94) |
| **Artistic** | 0.31 (8) | 100 (8) | 0 (0) |
| **Economic** | 2.08 (53) | 79.25 (42) | 20.75 (11) |
| **Sports** | 0.78 (20) | 80 (16) | 20 (4) |
| **Military and marine** | 0.71 (18) | 50 (9) | 50 (9) |
|  |  | 79.83 (2038) | 20.17 (515) |
